# Investing in breastfeeding – the world breastfeeding costing initiative

**DOI:** 10.1186/s13006-015-0032-y

**Published:** 2015-02-23

**Authors:** Radha Holla-Bhar, Alessandro Iellamo, Arun Gupta, Julie P Smith, Jai Prakash Dadhich

**Affiliations:** International Baby Food Network (IBFAN) Asia, BP 33 Pitampura, New Delhi, India; IBFAN Asia, 4535a Casino St, Palanan Makati, Metro Manila Philippines; Australian National University, Canberra, Australia; Breastfeeding Promotion Network of India, BP 33 Pitampura, New Delhi, India

**Keywords:** Breastfeeding, Costs and cost analysis, Breastfeeding investment, Child survival, Health plan implementation, Health promotion/Economics/*Organization & administration, National health programs/economics/*Organization & administration, Program development/Economics

## Abstract

**Background:**

Despite scientific evidence substantiating the importance of breastfeeding in child survival and development and its economic benefits, assessments show gaps in many countries’ implementation of the 2003 WHO and UNICEF *Global Strategy for Infant and Young Child Feeding (Global Strategy).* Optimal breastfeeding is a particular example: initiation of breastfeeding within the first hour of birth, exclusive breastfeeding for the first six months; and continued breastfeeding for two years or more, together with safe, adequate, appropriate, responsive complementary feeding starting in the sixth month. While the understanding of “optimal” may vary among countries, there is a need for governments to facilitate an enabling environment for women to achieve optimal breastfeeding. Lack of financial resources for key programs is a major impediment, making economic perspectives important for implementation. Globally, while achieving optimal breastfeeding could prevent more than 800,000 under five deaths annually, in 2013, US$58 billion was spent on commercial baby food including milk formula. Support for improved breastfeeding is inadequately prioritized by policy and practice internationally.

**Methods:**

The *World Breastfeeding Costing Initiative* (WBCi) launched in 2013, attempts to determine the financial investment that is necessary to implement the *Global Strategy*, and to introduce a tool to estimate the costs for individual countries. The article presents detailed cost estimates for implementing the *Global Strategy*, and outlines the WBCi Financial Planning Tool. Estimates use demographic data from UNICEF’s *State of the World’s Children 2013*.

**Results:**

The WBCi takes a programmatic approach to scaling up interventions, including policy and planning, health and nutrition care systems, community services and mother support, media promotion, maternity protection, WHO International Code of Marketing of Breastmilk Substitutes implementation, monitoring and research, for optimal breastfeeding practices. The financial cost of a program to implement the *Global Strategy* in 214 countries is estimated at US $17.5 billion ($130 per live birth). The major recurring cost is maternity entitlements.

**Conclusions:**

WBCi is a policy advocacy initiative to encourage integrated actions that enable breastfeeding. WBCi will help countries plan and prioritize actions and budget them accurately. International agencies and donors can also use the tool to calculate or track investments in breastfeeding.

## Background

More than 800,000 under five deaths a year could be prevented globally by achieving optimal breastfeeding practices, as recommended by the World Health Organization (WHO) and UNICEF – initiation of breastfeeding within the first hour after the birth; exclusive breastfeeding for the first six months; and continued breastfeeding for two years or more, together with safe, nutritionally adequate, age appropriate, responsive complementary feeding starting in the sixth month [1].

Global optimal breastfeeding rates remain abysmally low regardless of overwhelming scientific evidence to support the importance of optimal breastfeeding practices for child mortality, morbidity and malnutrition, and non-communicable diseases in adult life [2-5]. Equally, over the past decade, growth in global sales of baby foods mainly milk formula accelerated from $22.4 billion in 2003 to over $58 billion in 2013 [6]. Nearly half this sales growth has been in the developing countries of the Asia Pacific region, with breastfeeding declining rapidly in populous low and middle income countries such as China and Indonesia.

Health agencies have identified evidence-based strategies for promoting optimal breastfeeding in the 2003 WHO/UNICEF *Global Strategy for Infant and Young Child Feeding* (*Global Strategy*) [1]. The absence of economic perspectives has been cited as a barrier to the practical implementation of the Global Strategy [7], economic and financial factors are crucial to its justification and success. Markets fail to provide incentives for the achievement of optimal breastfeeding [8] and do not adequately promote and protect the associated potential for large international economic gains [9]. These economic and financial benefits for health systems in developed and developing countries have been demonstrated at both macro and microeconomic level [10-15]. Breastfeeding is one of the most cost effective ‘interventions’ to prevent under -five mortality [16]. Of available interventions, counselling about breastfeeding (and fortification) is said to have the greatest potential to reduce the burden of child mortality and morbidity [17]; breastfeeding programs cost from $100 to $200 per death averted, making them equally or more cost-effective than measles and rotavirus vaccination [18].

The *World Breastfeeding Trends Initiative* (WBTi) was established in 2004 to contribute to the monitoring and effective implementation of the *Global Strategy* with indicators to address its key policies and programs (Table 1) [19], The WBTi database currently includes 54 of the 75 Millennium Development Goal ‘countdown’ countries (where more than 95% of maternal, newborn, and child deaths occur). A recent study has used WBTi data on policy performance to show an association between aggregate scores and changes in exclusive breastfeeding (EBF) rates for 22 countries, suggesting that implementing *Global Strategy* policies and programs can increase EBF [19].

**Table 1:** **World Breastfeeding Trends Initiative (WBTi) policy and process implementation indicators**

**Indicators**	
1.	National policy, program and coordination
2.	Baby friendly hospital initiative (BFHI) (Ten steps to successful breastfeeding)
3.	Implementation of the WHO International code of marketing of breastmilk substitutes (International code)
4.	Maternity protection
5.	Health and nutrition care system (in support of breastfeeding and infant and young child feeding)
6.	Maternal support and community outreach/community-based support for the pregnant and breastfeeding mother
7.	Information support
8.	Infant feeding and HIV
9.	Infant feeding during emergencies
10.	Mechanisms of monitoring and evaluation systems

Gupta et al. reviewed the evidence and recommended that seven strategic actions need to be taken by countries to ensure good implementation of the *Global Strategy* [20]. A 2013 UNICEF report has identified, lack of political will and low financial investments for breastfeeding are contributing to the lack of progress in optimal Infant and Young Child Feeding (IYCF) [21]. For example, a recent report on the global status of breastfeeding policies and programs in 40 countries, using the WBTi database, showed that no country assessed has fully implemented the *Global Strategy* [22].

Creating an enabling environment for optimal breastfeeding requires full implementation of the *Global Strategy* including provision of unbiased correct information, protection from commercial pressures and misinformation through effective implementation of the WHO International Code of Marketing of Breastmilk Substitutes (henceforth the International Code) [23] and national legislation. Also important are the facilitation of establishment and referral to an effective support structure that includes one-to-one and group counselling, and adequate maternity leave protection to every woman, all of which need to be scaled up to 100%. While facilitating optimal breastfeeding requires financial outlay the health costs of premature weaning are increasingly evident [24-26], there is no corresponding investment to support women to this end and breastfeeding remains amongst the most under-funded nutrition interventions [27].

The World Bank’s *Scaling Up Nutrition* estimates [28] are widely used as a reference for costing including on nutrition interventions such as fortification or supplementation. These estimates of financial resources needed for scaled up implementation of maternal and child nutrition initiatives are a good start, but have insufficient detail to support a policy focus on breastfeeding. The estimates address just one component of the environment needed for making breastfeeding more universal – the promotion of behavioural change via counselling. Consideration of broader economic aspects could further the implementation of the policies and interventions in the Global Strategy [7].

The WBC*i* was launched in 2013 by International Baby Food Action Network (IBFAN) Asia and Breastfeeding Promotion Network of India (BPNI) to redress deficits in financial information needed to support implementation of the *Global Strategy.* The WBC*i* consists of an advocacy document, *The Need to Invest in Babies*, and a financial planning tool. While “The Need to Invest in Babies” provides estimates [29], the financial tool provides for ‘hands-on’ budgeting.

While the understanding of “optimal” breastfeeding may vary among, and within countries, there is a need to encourage governments to facilitate an enabling environment for women to achieve optimal breastfeeding.

This paper aims to present key information about the WBC*i* initiative, and to summarize findings about the potential cost of implementing the *Global Strategy* more widely.

## Methods for costing of interventions

### Data, scope, and costing approach

WBC*i* takes a programmatic approach to scaling up interventions in 214 countries, basing costs on either national amounts allocated to the intervention or on globally accepted costs. The costings are of direct, mainly financial costs from a governmental perspective [30-32]. All financial estimates are US dollars.

The estimate of financial outlays includes:

- One-off costs such as developing IYCF policies, and legislation on the International Code, and;
- Recurring costs such as: training of health workers and community volunteers in skilled counselling and implementing the International Code, media campaigns, maternity protection and monitoring of implementation.

#### Unit cost and total cost calculations

For calculating the unit cost for each intervention, the following steps were taken:

1. The unit cost was calculated for the country, that is, the data source.
2. The unit costs for the Baby Friendly Hospital Initiative (BFHI), mass media communication, legislating the International Code and community support for breastfeeding/IYCF have been taken from available published data (see Table 2). Where no published data was available, the median of costs budgeted by countries was computed based on information from colleagues and officials in relevant ministries in Brunei Darussalam, China, Egypt, Fiji, India, Mongolia, Saudi Arabia, Rwanda, Brazil and Australia.
3. Unit costs were adjusted for inflation to 2012 using the published World Bank inflation rates [33].
4. The inflation adjusted unit cost was converted into US dollars.^i^
5. Some costs were converted into international dollars using the World Bank purchasing power parity (PPP) conversion factors for 2012 [34]. These items were: 1) breastfeeding training for health workers; 2) community counselling incentives; 4) media promotion, and; 5) BFHI implementation.
6. Other costs, for policy development and review, drafting of a national code, legislation development and maternity entitlements, were computed using the currency exchange rate.
7. The PPP was available for 167 countries; where unavailable the local currency was converted into US dollars at the annual exchange rate.
8. The total cost for the intervention was calculated by the multiplying the unit cost by the global birth cohort, for 194 countries in the *State of the World’s Children 2013 *(*SOWC* [35]).
9. The price per child was derived indirectly using the adjusted unit cost. Data on the birth cohort is taken from *SOW* [35]. (While UNICEF data lists only 194 countries, the remaining 20 countries and territories are small, and birth cohorts are unlikely to be large relative to other included countries. We anticipate that the difference in total costs and cost per live birth will be minimal).
10. For calculating the estimate on maternity entitlements, we have allocated a minimum of $2/day per target recipient. This was done keeping in view the minimum sum required to survive as per World Bank estimates [36]. The number of households under the poverty line is estimated from *SOWC* [35].

**Table 2:** **Global financial resources creating the enabling environment for optimal breastfeeding**

**Total target (live births only) 214 countries**			**135,000,000**				
**Total population**			167 countries				
**No. of women living below the poverty line**			35,200,684				
**Country with legislated international code**			[33]				
**Total number of countries**			214				
**% of Births assisted in health facility**			61%				
***Description***		**What is included in the cost**	**Unit**	**No. of Units**	**Total Cost of 214 countries ($)**	**Cost per live birth $**	**Reference**
**One-time costs**							
1	*IYCF Policy development and review*	1. Meetings	Country	214	5,350,000	$0.04	Median Cost of 3) countries:
		2. WBTi review and analysis from IBFAN with discussions					1) Afghanistan
		3. Data processing and analysis					2) Fiji
		4. Consultations and drafting sessions					3) Mongolia
		5. Consultant					
2	*International code*	Drafting and Legislative process	Country	181	9,050,000	$0.07	Median Cost, four (4) countries: 1) China 2) Egypt 3) Fiji 4) Afghanistan
		1. Meetings					
		2. Discussions					
		3. Consultations and drafting sessions					
		Legislative Process	Country	181	470,600,000	$3.49	[37]
		1. Parliamentary/congress/ legislative process (sessions, committees and plenary debates)					
				***Subtotal 1***	***485,000,000***	***$3.60***	
**Annual cost (Recurrent) (Ministry of health, nutrition)**							
3	*BFHI Implementation*	1. Bed in	Country	214	2,010,000,000	$15	[38], adjusted to International US $
		2. Health education to mothers					
		3. No formula in the facility					
4	*Training of health workers*	1. Breastfeeding training for health workers (nurses, midwives)	Country	214	251,000,000	$2	[39]
5	*Community support*	Incentive and training for community volunteers	Country	214	1,340,000,000	$10	[28]
6	*Media promotion*	Cost of media (radio) advertising	Country	214	723,000,000	$5	[40]
7	*Training on the international code*	Five (5) day training on 1) understanding and 2) monitoring the International Code	Country	214	11,769,615	$0.10	India training experience
8	*Monitoring*	Monitoring the implementation of the different programs (International code, BFHI, community)	Country	214	81,000,000	$0.60	[28]
				***Subtotal 2***	***4,414,800,000***	***$33***	
**Annual cost (Ministry of labor, social security system ministry of finance, Ministry of social welfare**							
9	*Maternity entitlements*	Allowance for women living below 2 $/day for a period of six months	Country	214	12,700,000,000	94	2U$/day as a minimum entitlement based on the poverty line set by World Bank
				***Subtotal 3***	12,700,000,000	94	
				**Total**	**$17,601,800,000**	**130**	

#### Overhead and capital infrastructure costs excluded

The necessary delivery platforms for the *Global Strategy* are the following:

- Health facilities;
- Community health and nutrition programs and home deliveries;
- Mother support groups/family level communication.

We have not included overhead costs (for example, administration salaries) or capital costs of delivery systems for health services and other social and economic infrastructure in our estimates. These differ widely from country to country, and countries are assumed to have this basic delivery capacity.

The public sector health and the nutrition delivery system, with its community outreach programs that reach out to families regularly for antenatal check-ups, distribution of iron and folic acid tablets, tetanus toxoid injections during pregnancy, weighing and growth monitoring of infants and young children and immunization, are already equipped with human resources and transport facilities. We assume that with an additional component of training in skilled counselling, this cadre of workers will deliver the required services.

It is assumed that the government health and nutrition sector and the general public will monitor implementation of the International Code and the regulatory machinery of the government will have enforcement capacity. For provision of maternity benefits, the costings assume that various sectors of the governance system – health and nutrition sector, labor, welfare sectors – as well as the general public will monitor the implementation of maternity legislation and ensure that action is taken against violations.

Finally, the government is taken to administer the disbursal of financial benefits through existing structures and systems adapted to local conditions, at zero or minimal additional cost.

#### Non market economic goods and services excluded

Considering community based or volunteer mother-to-mother support groups, we have assumed the existence and capacity of community volunteer groups, who will be given training and some financial incentives for counselling, but we have not fully budgeted most costs, or the organizational costs for developing, maintaining and expanding breastfeeding support groups. (The value of these volunteer contributions may be several times the financial contribution from government, based on unpublished estimates for Australia). We did not estimate the economic cost to women of additional time spent breastfeeding.

We estimated costs for the following categories of action. Table 2 sets out details of the costings in these categories.

### Main cost categories

#### Development of optimal breastfeeding strategies, policies and plans, and coordination

Effective policies are based on adequate planning and policy development processes, and coordination of relevant policies and services. The *Global Strategy* is presented as an integrated and comprehensive program, and such an approach has been found to be effective in achieving better breastfeeding practices [41]. In consequence the WBC*i* costing include the costs of hiring national and international consultants, holding workshops and consultations, developing documents, building consensus, printing and dissemination, multi-sectorial coordination and regular review and analysis of the progress made in implementing the agreed plan using the *WBTi* tool. The estimate is based on the unit cost worked out as a median of the costs of this activity in Afghanistan, Fiji and Mongolia. While policy making is treated as a one-off cost, monitoring and review are considered annual recurrent costs.

#### Implementing the international code of marketing of breastmilk substitutes

An important element of the *Global Strategy* is to constrain inappropriate marketing and consumption of breast milk substitutes (BMS) including milk formula for infants and toddlers. In response to concern about unregulated marketing of BMS and potential detrimental effects on infant and child mortality, the International Code was adopted at the World Health Assembly in 1981 by 118 member states. A recent review by WHO found that in 2012 only 37 of 199 countries (19%) had fully implemented the International Code including by legislation [42].

The WBCi estimate includes three components – drafting the law, law making and training in International Code implementation. While the former two are one-time costs, the latter is a recurring cost. The estimate covers the cost of hiring national and international consultants, holding workshops and consultations, advocacy and building consensus, cost of the passage of the law through the legislature and training a cadre of government officials in recognizing violations, monitoring adherence and initiating action in case of violations. The estimate does not include the costs involved in any legal or judicial action in the case of violations.

#### Drafting national legislation to implement the international code

This estimate is the median of the costs incurred in Afghanistan, China, Egypt and Fiji.

#### Law making to implement the international code

In 2012 Wilson and colleagues estimated based on a study in New Zealand, that $2.6 million is needed to pass a public health law [37]. We have used this figure as the unit cost of legislating the International Code.

#### Training for officials and others on the National Code/Legislation

The one-off costs of training of 200 officials over five years is based on 2-day training workshops held in India, at $23,160 per training (actual cost of training workshops). The costs of training field level workers was not estimated, it was assumed that the skill training course provided will include a component on the International Code (national measure). For subsequent monitoring of violations we have used the estimation by Breastfeeding Promotion Network of India of $1927 per district with a population of 1–2 million [43].

#### Baby Friendly Hospital Initiative (BFHI) implementation, and health worker training

The health care system itself has been used as an avenue to promote formula feeding and undermine breastfeeding, and recent studies point to targeting of health workers by industry. Recent media reports have highlighted corrupt and unethical promotion of formula feeding through hospitals and health professionals in China [44,45]. This has also been demonstrated historically for Australia [46], and in the Philippines where a recent study by WHO found that health workers were targeted by company promotions, and these were highly influential in leading mothers to initiate formula feeding [47]. This WBCi cost category has two components, full implementation of the WHO/UNICEF BFHI, and training of health workers:

#### BFHI (Ten Steps to Successful Breastfeeding)

The cost includes developing a hospital policy, its dissemination, appropriate training of hospital staff (nurses, lactation counsellors), skilled counselling and support at birth and during the stay of mothers. The unit cost is based on the results from the study conducted through maternity services in Brazil, Honduras and Mexico for estimating the costs and impact of breastfeeding promotion programs [48]. The suggested costs to eliminate infant formula, to practise rooming-in and the cost per birth for maternal education activities within the health facility, including their training were combined. The cost per birth assisted in a health facility (61%), was used to estimate the costs for health facilities with maternity services to implement the 10 steps for successful breastfeeding.

#### Training public health workers

The unit cost was calculated from the cost of replicating the LINKAGES LAM Promotion Program in Jordan from December 2001–2002 [39], and was adjusted for inflation. The estimate includes costs of training of midwives, public health nurses and other health workers who conduct deliveries at homes and provide counselling and other services as part of the health system’s services.

#### Community services and mother-to-mother support

Practical and well informed support for breastfeeding in the community is crucial and cannot be taken for granted. The WBCi estimate uses costs of such community services as calculated by Mason [49], adjusted for inflation to 2012. It includes training of field workers (community based/peer counsellors), volunteers and mother support groups providing counselling services at the household level in the community, as well as some incentives provided from the health and nutrition system. We have further included a unit cost per live birth for refresher courses, based on actual expenses incurred by the BFCHI Project in Lalitpur, India.^ii^

#### Media promotion/social marketing

The WBCi estimates include the cost of breastfeeding promotion programs using mass and local media to counter industry messages undermining breastfeeding on an on-going basis (‘social marketing’) [50]. The general principle should be that investment in breastfeeding promotion should match that by the industry in the same market; effective enforcement of the International Code through regulation would help lower such government ‘social marketing’ costs [51]. A typical rule of thumb for industry is a marketing expense of 10% of gross sales value [52], more in highly competitive industries such as pharmaceutical industries, less in stable markets [53]; an estimate in this range is supported by a recent WHO study in the Philippines [54]. We have therefore estimated the cost of radio campaigns at $5 per child, as recommended in the World Bank’s report No. WPS 952 in 1992 [40]. The amount per child has been adjusted for inflation to 2012.

#### Monitoring

This estimate includes the cost of monitoring the implementation of the interventions, including development of the national policy and plan of action, the national law that implements the International Code, BFHI, etc., review meetings, updating of policies and plans and operational research. The estimate in *Scaling Up Nutrition* of $200 million for the monitoring and evaluation of interventions that target 326 million children, is used to compute the cost per beneficiary. As most countries are already conducting national surveys on breastfeeding practices, this cost was not taken into account.

The financial resources needed for conducting national assessments of implementation of the *Global Strategy* using WBT*i* were estimated [55]. Currently 82 countries from Asia, Latin America, the Caribbean and Africa are utilizing this tool to track and monitor their implementation of the *Global Strategy* and identify gaps. The cost per country for using this tool and building consensus over the assessment and identification of gaps, developing report cards and national reports, is about $4000, which includes country cost of minimal expenses and project costs at the regional level.

#### Maternity leave protection

An important non-market economic cost of breastfeeding is the potential economic opportunity cost of the mother’s time spent exclusively breastfeeding [56-59]. This points to the importance of paid maternity leave policies, which have been shown to correlate with substantially higher breastfeeding rates in a recent global study [60]. In the WBC*i*, a flat rate of $2 per day/mother for 180 days is costed as financial assistance for women living below the poverty line. Providing a woman with this amount is assumed to offset some economic costs to the mother and her family by enabling her to stay in necessary proximity to her newborn for longer with six months of exclusive breastfeeding. We have not computed the costs of provision of maternity benefits for women working in the formal sector as these vary widely from country to country, both in amount and in the source of financing. We have not estimated the cost of setting up crèches, or of workplace accommodations needed by employed mothers of infants and young children to ensure their proper care and feeding.

## Results

### Global costs

Table 2 gives the estimated cost per intervention and aggregate costs.

Using the WBCi costing tool, the cost of implementing the *Global Strategy* program for 214 developing countries is estimated at $17.6 billion.

### Recurring costs

Importantly the major recurring cost is maternity entitlements. The global cost of implemented a minimum entitlement based on the World Bank poverty line is $12.6 billion. This highlights that initiatives to protect, promote and support breastfeeding will need to address the economic implications for women to be successful. While breastfeeding has less financial cost to the mother, the need for proximity to the infant and for ‘breastfeeding friendly’ workplaces and childcare, can be a barrier to maternal employment and income earning activities. As employment and childcare arrangements may be inflexible to the needs of breastfeeding mothers, paid maternity leave policies and related measures are increasingly essential for countries experiencing rapid economic growth and employment opportunities.

### Other major costs

Other major cost items are implementation of the BFHI which accounts for $2.1 billion of the total cost, and Community Support which accounts for $1.3 billion of this cost.

## Discussion

The WBCi is a landmark first attempt to cost the implementation of the *Global Strategy* in its entirety and integrate it into other global nutrition initiatives notably the World Bank initiative, *Scaling Up Nutrition* [28]. The World Bank report noted the staggering cost of under-nutrition worldwide, and the loss of billions of dollars in foregone productivity and avoidable health care spending. The public funding resources of $11.8 billion annually said to be needed to counter the problem included increasing breastfeeding as one of the 13 proven nutrition interventions. However, these costings made inadequate provision for breastfeeding, providing only for programs of ‘behaviour change’ but without addressing the key structural barriers to optimal breastfeeding, especially the time pressure and labour market barriers to breastfeeding, and the health services, community support, and marketing issues identified by the *Global Strategy* and related initiatives.

The WBCi focusses on redressing such costing gaps, and the major finding of the global analysis using the WBCi tool is that the financial cost of implementing the WHO/UNICEF *Global Strategy* for 214 developing countries is in line with the cost of such major global nutrition initiatives. One-off costs of initiating implementation globally are around $485 million, while the recurrent costs including maternity protection are $17.1 billion. Other significant costs are associated with BFHI implementation, and improving community based support for breastfeeding.

The WBCi costing is likely to underestimate actual costs of implementing the *Global Strategy* in its entirety worldwide, as unit costs have been based on the few available estimates, mainly from developing countries; these estimates are clearly lower than if the same interventions were budgeted for in developed countries which are likely to have higher costs. On the other hand it may overestimate costs if a strong regulatory approach is taken. For example, costs of social marketing expenditures and health worker training could be minimized by strong legislation addressing inappropriate marketing and promotion of breastmilk substitutes.

The accuracy of the estimates is clearly most sensitive to assumptions for the three major cost areas. For example, raising the level of the maternity entitlement by 20% to $2.40 a day would add some $US2.5 billion to the $US17.6 billion cost. Likewise, if BFHI implementation unit costs were taken to be $US10 rather than $US15 (for example, to partly account for potentially offsetting lower current and future health system costs), the cost of BFHI implementation would be reduced by $US670 million to around $US1.3 billion. Increasing the budget for incentives and training for community volunteers (the third largest cost item) by 20% would add just $US270 million to the overall cost.

The cost analysis also provides a figure that draws attention to the offsetting benefits of improved breastfeeding rates. Several economic studies have shown the high economic value of breastfeeding, both in reducing burdensome family expenditures on breast milk substitutes or avoidable health costs, and in increasing the production of human milk [10-13,15,18,24,25,57,61-63]. At prices currently paid in developed countries of over $85 per litre, the global economic value of breast milk production, if optimal breastfeeding were achieved for 95% of infants and young children, has been estimated to be more than $3,380 billion a year [9]. Using the methodology in such studies, this suggests an economic gain of nearly $1,400 billion a year comparative to current levels. By comparison with the financial outlay to implement the *Global Strategy*, the potential economic and other benefits of improved breastfeeding rates are high.

The analysis highlights also that as no country has yet substantially implemented the *Global Strategy*, or budgeted for its implementation, little has been done worldwide to facilitate optimal breastfeeding.

The fundamental premise of this paper is that access to interventions to achieve optimal IYCF practices (breastfeeding and complementary feeding) is a right of every woman and child [64]. Each woman who gives birth requires an enabling environment to achieve optimal breastfeeding. Our study focuses on financial costs to the government sector and does not directly account for personal or household costs of breastfeeding which may include foregone maternal earnings, career opportunities, or other maternal constraints.

There are wider benefits for maternal and child health and well-being from an initiative for maternity entitlements, beyond breastfeeding, such a program would help address poverty among women as a source of inequitable access to optimal breastfeeding. Program benefits could be reinforced by labour market regulations giving employers and childcare services appropriate incentives to support optimal breastfeeding amongst employed mothers. This approach acknowledges the real economic costs and time constraints of optimal breastfeeding to households and to women in particular: as noted earlier, research in developed and developing country settings has shown that exclusive breastfeeding can be very time costly if appropriate support for lactation is not available in work environments and childcare services.

### Strengths and limitations

The unique strengths of this study and the research that underpins it are that it provides both conceptual and practical advances which can inform global, country level, and community efforts to implement the *Global Strategy.* This includes its strong and comprehensive conceptual basis:

1. being structured around evidence based interventions that would contribute to the improvement of optimal breastfeeding practices;
2. based on strategies and interventions that government and developmental partners are implementing at the national and sub-national level;
3. contributing a novel initial framework to estimate the resources necessary to be invested to implement the above;
4. Conceptually comparable with estimates of the economic value of breastfeeding, and the health system benefits of optimal IYCF.

Its other strengths are that its design and development includes a platform to share country experiences on investing and an opportunity to identify data/information gaps that may need to be addressed with more research in the area. The research further contributes by providing a priority research agenda on IYCF for initiatives by international and national agencies such as on maternal and child nutrition. It can also help the global community to move beyond the need to invest on commodities and to include programmatic aspects in their investments.

Limitations of our study include that up to date research to underpin program level cost estimates on reducing suboptimal IYCF is sadly lacking; there is an urgent need to update and extend previous economic studies on interventions to increase breastfeeding such as those by Horton and colleagues [40,48]. The World Bank has provided strong standardized unit cost data for IYCF promotion in *Scaling Up Nutrition*; however, more detailed cost data from recent fieldwork is crucial for implementation of the range of known effective interventions for increasing breastfeeding.

Apart from this major consideration, the financial resources estimated in this document have the following specific limitations:

1. Lack of data on birth cohorts and number of households below the poverty line in 20 countries and territories.
2. Wide divergence between staff responsibilities, salaries, transport costs and infrastructure costs among nations. Thus, while the interventions require an increase in human resources and the resultant financial resources in most countries, we did not attempt to cost either the number of staff required nor staff salaries.
3. Limited information on the kind of maternity protection and maternity entitlements that are being offered to women working in the unorganized/informal economy, as well as to homemakers in households below the poverty line, in several countries. Also lack of robust information on any incentive effects on maternal labour force behaviour that would need to be included in a full economic costing model.
4. The costs of other direct and indirect interventions that impact optimal breastfeeding practice, such as food supplementation for mothers and children, including micronutrients and foods for preventing and managing malnutrition, special needs of infants in the context of HIV/AIDS, were not estimated.
5. Our estimates are also fundamentally constrained by cost estimates being from the health agency/government perspective. While limited maternity protection costs are included, the perspective taken excludes the unpaid household sector, and therefore in particular, any additional economic costs to mothers, or to those working for volunteer organizations and groups providing necessary services and activities underpinning community based, mother to mother support for breastfeeding.
6. Assuming constant returns to scale. There may be economies of scale or the program may get more complex as it is scaled up.

### The WBC*i* financial planning tool

The WBCi costing analysis also highlights the need for countries to estimate more accurately the costs of implementing the *Global Strategy* in their context and illustrates the importance and potential value of using the WBCi Financial Planning Tool for country specific analysis.

The WBCi, a milestone initiative of IBFAN Asia, has been developed following the principles and structure of the WBT*i* tool to help countries track the implementation of the *Global Strategy* and identify gaps in its implementation. The WBCi tool is a user- friendly MS Excel tool to help countries comprehensively estimate the resources necessary to implement the policy and programs proposed by the *Global Strategy,* as well as specific detailed plans to bridge the gaps identified by WBTi and budget them adequately and accurately according to local conditions. It can be used to generate annual IYCF financial plans, multi-year estimates, and budget proposals, using local estimates, inputs and information.

The WBC*i* Financial Planning Tool [65] can be used to conduct studies at the individual country level and global level. It can be contextualized at the country level through simple key parameters, such as exchange rates and unit costs or salary levels, chosen by the user.

The WBC*i* tool is intended for national program managers and partners to initiate a constructive and productive advocacy with national governments and donors, towards the identification and allocation of the financial and human resources necessary to protect, promote and support optimal breastfeeding and complementary feeding practices in the country. It may also be used to track budgets.

The key features of the tool include the following:

- Customization of the cost, using the most recent national data;
- Generation of individual cost for the key interventions recommended by the *Global Strategy*;
- Tracking estimates *vs.* actual resources provided by government and donors;
- Generation of reports for discussions and presentations to other stakeholders;
- Review and update cost estimates based on program monitoring and evaluation.

The WBCi tool has been successfully used by Department of Nutrition, Ministry of Public Health, Afghanistan, to budget and raise funds for specific actions such as celebration of the World Breastfeeding Week, as well as broader programs such as training of health professionals and field workers in counselling skills.^iii^

## Conclusions

The WHO’s scientific analysis of benefits of breastfeeding on child health and development, extending well into adult life and increasing IQ, cannot be ignored, nor can the evidence of its impact on reducing infant and child mortality and malnutrition, and its importance for maternal reproductive health. However, demonstration of the budgetary feasibility and sustainability and potential economic gains from the *Global Strategy* is important to its practical implementation. The time has come to transform the token attention breastfeeding often receives into a non-negotiable commitment to the full implementation of the *Global Strategy* through multi-sectoral action, rather than the implementation of only a few interventions.

In order to breastfeed successfully, women must have access to all the services that protect, promote and support breastfeeding. Women need to be physically close to their infants and to be confident about their ability to feed their infants adequately. They should also be free from pressure by the milk formula manufacturers so as to make good infant feeding decisions. This may require strict enforcement of the International Code of Marketing of Breastmilk Substitutes and/or national laws, and behaviour change that can be achieved through skillful counselling, ideally during pregnancy and at family level. Interventions that provide protection from commercial sector competition and support at health facilities and in the workplace are vital.

The ‘market’ and the incentives it creates cannot be relied on to protect, promote and support breastfeeding, as is well illustrated by economic analysis of ‘market failures’ in this area [7,8]. Governments and international agencies have a responsibility to ensure resources commensurate with the benefits of optimal breastfeeding. Global and national attention should be visible, especially in terms of making financial resources available within and to countries. With the problem of malnutrition increasing worldwide as obesity adds to problems of under-nutrition, decisive action on IYCF is urgently needed.

The following is a set of recommendations to move forward:

### Governments (and relevant agencies as appropriate) should

1. Plan and budget for the comprehensive global and national implementation of the *Global Strategy*, and integrate its implementation as part of national development and economic priorities.
2. Conduct policy and program assessments on breastfeeding and IYCF using WHO assessment tools or WBT*i* tools in order to identify and document gaps.
3. Develop or assist development of national and sub-national action plans for 1–5 years with clear budgets to achieve results, based on the policy gaps found.
4. Develop or assist development of national/regional/provincial-monitoring and periodic reporting systems on optimal breastfeeding practices.
5. Institutionalize research to document benefits of this program to populations, in terms of disease reduction and long term health as well as cost savings.
6. Report annually on the key expenditures incurred on interventions for optimal breastfeeding and track intervention progress, in all major areas of action noted above.
7. Take urgent action on policy matters such as maternity leave and other measures.

### The global community should

1. Allocate specific budgets for increasing optimal breastfeeding within existing global funds for child survival, nutrition and health (All donors and global agencies).
2. Revisit estimates on *Scaling Up Nutrition,* giving full consideration to all interventions required for universal services to facilitate optimal breastfeeding. (World Bank).
3. Make a priority commitment for universal application of the International Code and IYCF (WHO, UNICEF, World Bank).
4. Report annually the money spent on programs on improving policy and programs for optimal breastfeeding (All agencies).
5. Setup a special maternity benefit fund for cash assistance to women below the poverty line (World Bank).

## Endnotes

i. “An international dollar is hypothetical currency that is used as a means of translating and comparing costs from one country to the other using a common reference point, the US dollar. An international dollar has the same purchasing power as the US dollar has in the United States. Costs in local currency units are converted to international dollars using purchasing power parity (PPP) exchange rates” (source: WHO CHOICE: http://www.who.int/choice/costs/prog_costs_intro/en/
ii. Personal communication from Dr. K.P. Kushwaha, 19 September 2012.
iii. Personal communication, Dr H Ludin, Ministry of Public Health, Afghanistan.
